# Control of proliferation in the haploid meristem by CLE peptide signaling in *Marchantia polymorpha*

**DOI:** 10.1371/journal.pgen.1007997

**Published:** 2019-03-07

**Authors:** Yuki Hirakawa, Naoyuki Uchida, Yasuka L. Yamaguchi, Ryo Tabata, Sakiko Ishida, Kimitsune Ishizaki, Ryuichi Nishihama, Takayuki Kohchi, Shinichiro Sawa, John L. Bowman

**Affiliations:** 1 Department of Life Science, Faculty of Science, Gakushuin University, Tokyo, Japan; 2 Institute of Transformative Bio-Molecules (WPI-ITbM), Nagoya University, Nagoya, Japan; 3 School of Biological Sciences, Monash University, Melbourne, Victoria, Australia; 4 Division of Biological Science, Graduate School of Science, Nagoya University, Nagoya, Japan; 5 Graduate School of Science and Technology, Kumamoto University, Kumamoto, Japan; 6 Graduate School of Biostudies, Kyoto University, Kyoto, Japan; 7 Graduate School of Science, Kobe University, Kobe, Japan; "USDA-ARS Pacific West Area", UNITED STATES

## Abstract

The homeostasis of meristems in flowering plants is maintained by cell-to-cell communication via CLE (CLAVATA3/EMBRYO SURROUNDING REGION-related) peptide hormones. In contrast, cell signals that regulate meristem activity remains elusive in bryophytes that maintain apical meristems in the gametophyte (haploid) body and undergo a gametophyte-dominant life cycle. We here show that MpCLE1 confines the proliferative activity of gametophytic meristem and affects the overall size of gametangiophores (reproductive organs) in *Marchantia polymorpha*, which is in sharp contrast with the meristem-promoting function of its ortholog TDIF/CLE41/CLE44 in Arabidopsis vascular meristems. Expression analysis suggests that Mp*CLE1* and its receptor gene Mp*TDR* are expressed in distinct patterns across the apical meristem. These data suggest that local CLE peptide signaling may have had a role in regulating cell proliferation in the shoot meristem in the ancestral land plant and acts in both sporophytic and gametophytic meristems of extant plants.

## Author summary

Land plants undergo an alternation of generations where both haploid and diploid phases develop multicellular bodies. Their growth relies on the activity of meristems at the growing tips of their bodies. Here we show a CLE peptide hormone acts as an intercellular signal controlling proliferative activity in the apical meristem of *Marchantia polymorpha*. Our finding reveals a general association of CLE peptide signaling with meristem homeostasis, a feature that evolved in the ancestral land plant, in both haploid and diploid phases.

## Introduction

Land plants have evolved unique peptide hormones to control various physiological processes including development and stress responses [1, 2]. A notable example is CLE (CLAVATA3/EMBRYO SURROUNDING REGION-related) family peptides involved in various developmental contexts in flowering plants, such as stem cell maintenance in meristems, vascular development, seed formation and growth control in response to environmental cues [3, 4]. The 12–13 amino acid CLE peptides are proteolytically processed from precursor proteins encoded by *CLE* genes [5–7]. CLE peptide hormones undergo post-translational modification such as proline hydroxylation and arabinosylation during maturation [8]. Mature CLE peptides bind to specific membrane receptors that transmit signals to direct cell behavior, thereby manifesting cell-to-cell communication [9, 10]. For example, the leucine-rich repeat receptor kinase CLV1 (CLAVATA1) is a receptor for the CLV3 peptide in Arabidopsis, participating in the stem cell homeostasis in the shoot apical meristem [11–13]. A phylogenetically related receptor, TDR/PXY (TDIF RECEPTOR/PHLOEM INTERCALATED WITH XYLEM), mediates TDIF (tracheary element differentiation inhibitory factor) peptide signaling essential for stem cell maintenance in the vasculature [14]. CLV3 and TDIF peptides possess characteristic residues for exclusive interaction with their specific receptors [15] and represent two major subclasses of CLE peptide family.

Comparative genomics studies have revealed that the repertoire of developmental regulatory genes is conserved among land plants even though body plans vary among different groups [16–18]. All land plants undergo alternation of generations where the both haploid (gametophyte) and diploid (sporophyte) phases develop multicellular bodies and one of the two phases is dominant depending on the plant lineage. Phylogenetically, the monophyletic, diploid-dominant vascular plants either nest within a bryophyte grade, or are sister to a clade of bryophytes that possess haploid-dominant life cycles.

**Fig 1:**
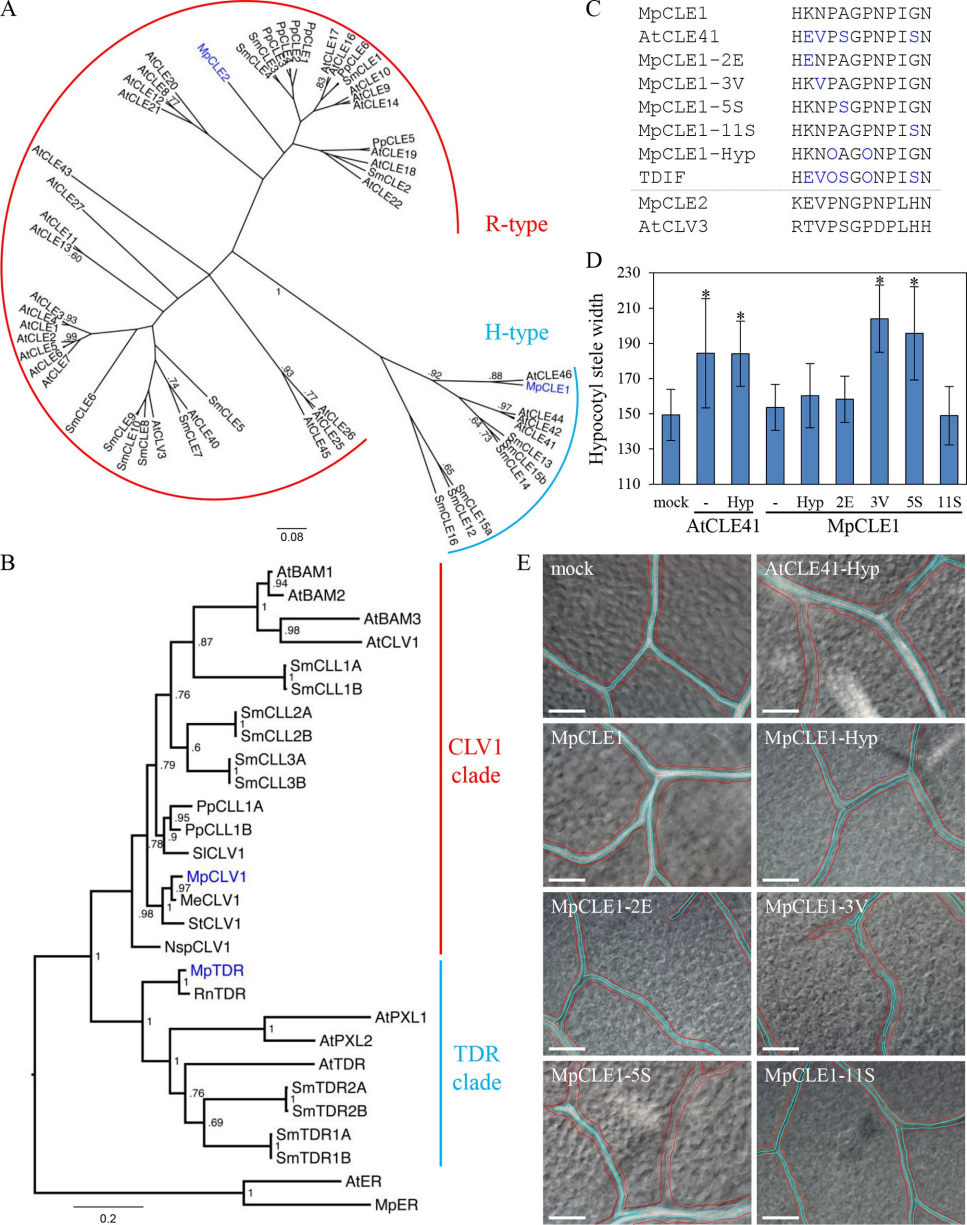
MpCLE1 peptide variants mimic Arabidopsis CLE41/TDIF. (A) A phylogenetic tree of CLE peptides based on their 12 amino-acid CLE peptide sequences. Two subgroups R-type and H-type are indicated. (B) A phylogenetic tree of CLE peptide receptors based on their kinase domain sequences. TDR and CLV1 subclades are indicated. ER(ERECTA) is set as the outgroup. The posterior probabilities of trees are shown at the nodes in (A) and (B). At, *Arabidopsis thaliana*; Sm, *Selaginella moellendorffii*; Nsp, *Nothoceros* sp.; Pp, *Physcomitrella patens*; Sl, *Sphagnum lescurii*; Me, *Marchantia emarginata*; Mp, *Marchantia polymorpha*; Rn, *Ricciocarpos natans*; St, *Sphaerocarpos texanus*. (C) Peptides used in the assays. Blue characters indicate residues different from the MpCLE1 peptide. Residues “O” indicate hydroxyprolines. (D) Effects of 10 μM peptide treatment on stele thickening in the hypocotyls of 10-day-old Arabidopsis. Peptides are indicated below. “Hyp” and “-” indicate the peptides with and without hyrdoxyprolines, respectively. Note that AtCLE41-Hyp is identical to TDIF. Data represent mean values ± s.d. (n = 13–16). Asterisks indicate a significant difference from mock treatment (0 M) in Welch’s *t*-test, p<0.05. (E) Effects of 10 μM peptide treatment in the leaf vein of 10-day-old Arabidopsis. Red and cyan lines indicate vein and xylem strand, respectively. Scale bars = 100um.

In both types of body plans, meristems function as the source of growth by continually providing new undifferentiated cells. This is achieved by functional zonation of meristems: one or few pluripotent stem cells act as a source of rapidly proliferating cells that often undergo specific division orientations giving rise to differentiating cells [19]. CLE peptides function in shoot, root and vascular meristems of Arabidopsis and other vascular plants, by controlling cell division and differentiation. TDIF, encoded by *CLE41/44* in Arabidopsis, is involved in three aspects of vascular cell behavior: inhibition of cell differentiation, enhancement of proliferation and control of cell division orientation [5, 20, 21]. *TDR/PXY* encodes a leucine-rich repeat receptor kinase (LRR-RK) of which extracellular LRR domain forms a superhelical structure that binds TDIF at its inner surface [8, 22–24]. We have previously reported that the TDIF activity in vascular development is conserved in most vascular plants [25]. However, the biological function of TDIF/CLE, as with any other peptide hormones, is poorly understood in bryophytes. An intriguing question is whether the TDIF/CLE peptides in bryophytes control the meristem activity in the gametophytic body. We here investigated the role for TDIF/CLE peptide in the liverwort *Marchantia polymorpha*, a model bryophyte species [26, 27]. Like many other developmental regulatory genes, the CLE gene family is conserved among land plants [28]. The *M*. *polymorpha* genome encodes two CLE genes, Mp*CLE1* and Mp*CLE2*, belonging to two distinct subclasses (H-type including TDIF and R-type including CLV3, respectively) of the CLE family based on the initial amino acid in the mature peptide hormone motif (Fig 1A, S1 Fig) [25, 29]. In addition, two distinct receptors for the CLE peptides, MpTDR and MpCLV1 are encoded in the *M*. *polymorpha* genome (Fig 1B) [29]. In contrast, the moss *Physcomitrella patens* has only R-type *CLE* genes and CLV1-type receptors. Thus, *M*. *polymorpha* provides a model system for studying TDIF/H-type CLE signaling in bryophyte development.

## Results

### Mp*CLE1* encodes a TDIF-type CLE peptide

To test if Mp*CLE1* is functionally equivalent to At*CLE41*, a TDIF-encoding gene of Arabidopsis, we generated gain-of-function alleles in Arabidopsis. The effects of constitutive TDIF expression in Arabidopsis have been previously observed [5, 15, 20, 30]. In the first leaves of 14-day-old seedlings, xylem vessels are formed along the leaf vein in wild type (S2 Fig) but it can be fragmented when At*CLE41* is driven by the constitutive *35S* promoter (S2 Fig; *35S*:At*CLE41*). In addition, hypocotyl stele thickening can be enhanced in *35S*:At*CLE41* plants compared to wild type (S2 Fig). Along with these vascular phenotypes, overall plant growth can be significantly reduced in *35S*:At*CLE41* plants (S2 Fig). In contrast, *35S*:Mp*CLE1* plants did not exhibit any of these phenotypes (S2 Fig) despite the expression level of Mp*CLE1* in this line being comparable to that of At*CLE41* in the *35S*:At*CLE41* line (S2 Fig). We further examined CLE bioactivities by peptide treatment assays [5, 31]. Treatment with 5 μM TDIF caused xylem fragmentation and stele thickening resembling At*CLE41* overexpression phenotypes (S2 Fig). In contrast, MpCLE1 peptide treatment did not induce these effects (S2 Fig). Similar results were obtained in assays with 20 μM peptide (S2 Fig). Collectively, these data indicate that MpCLE1 cannot functionally replace AtCLE41/TDIF in Arabidopsis.

To elucidate the discrepancy between the phylogenetic and functional relationships of MpCLE1 and AtCLE41, we performed amino-acid swapping between the two peptides, which differ at four residues (Fig 1C; MpCLE1 and AtCLE41). We synthesized four MpCLE1 peptide variants in which one of the four residues is changed to that of the AtCLE41 peptide (Fig 1C; MpCLE1-2E/-3V/-5S/-11S, respectively). In addition, hydroxyprolines were incorporated in an MpCLE1 variant (MpCLE1-Hyp) to mimic the natural structure of TDIF (Fig 1C; O indicates hydroxyproline). Among these MpCLE1-variants, MpCLE1-3V and MpCLE1-5S enhanced stele thickening (Fig 1D) and suppressed xylem differentiation in the leaf vein (Fig 1E). These data indicate that Mp*CLE1* is indeed a TDIF-type CLE gene although a minor amino-acid substitution is required to convert MpCLE1 into a functional peptide in Arabidopsis. Since the MpCLE1-type residues (Fig 1C; N3 and A5) differ from all other known CLEs in land plants, acquisition of these residues in MpCLE1 may have occurred in the liverwort lineage.

### MpCLE1 peptide suppresses growth of the *M*. *polymorpha* thallus

**Fig 2:**
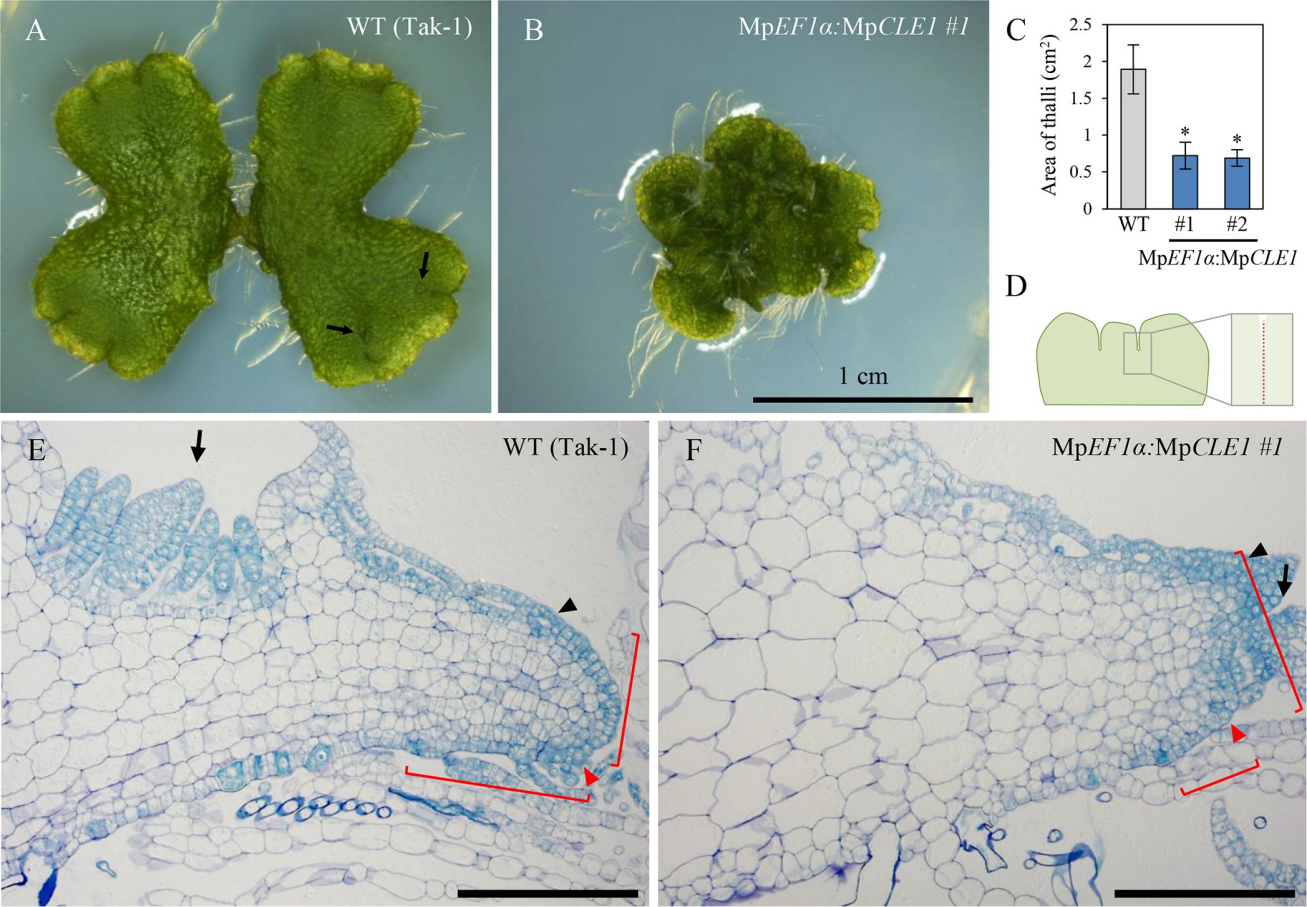
Gain-of-function phenotypes of Mp*CLE1*. Morphology of 14-day-old *M*. *polymorpha* plants grown from gemmae. (A and B) Overall morphology of (A) wild type (Tak-1) and (B) Mp*EF1α* promoter-driven Mp*CLE1*-overexpression line. (C) Area of thalli (mean ± s.d.). Two independent transgenic lines were used for quantification. Asterisks indicate a significant difference from WT in Weltch’s *t*-test (p < 0.001, n = 18). (D) Schematic diagram of section planes in apical notch. Longitudinal sections of apical notches in (E) wild type (Tak-1) and (F) the Mp*CLE1*-overexpression line. Arrows, red and black arrowheads, and brackets indicate developing gemmae cups, apical cells, developing air chambers closest to the apical cell, and proliferative region of meristem, respectively. Scale bars = 1cm in (A and B), 200 μm in (E, F).

We next analyzed the biological function of MpCLE1 in *M*. *polymorpha*. *M*. *polymorpha* is a thalloid liverwort and the body (thallus) grows at the apical notches which are indeterminate apical meristems and bifurcate periodically. For the clonal propagation, disc-shaped small progenies called gemmae are formed in gemmae cups that develop at the dorsal side of the thallus near the apical notch (Fig 2A). When mature gemmae were cultivated for 14 days on solid medium supplemented with MpCLE1 peptide, the overall growth of plants was slightly reduced, and thallus lobes were twisted (S3 Fig). Similar and marginally stronger phenotypes were observed with MpCLE1-Hyp peptide (S3 Fig). Unexpectedly, the effects of TDIF were even stronger than those of MpCLE1 peptides (S3 Fig). Transgenic lines overexpressing either Mp*CLE1* or At*CLE41* formed small and convoluted thalli (Fig 2A and 2B, S3 Fig). Quantification of the area of thalli showed significant reduction of growth in the Mp*CLE1* overexpression lines (Fig 2C). In addition, Mp*CLE1* overexpression lines produced fewer gemmae cups and did not form gametangiophores even 2 months after far-red light induction (S3 Fig).

To verify the minimal functional domain of MpCLE1, we produced overexpression lines with C-terminal deletions (Mp*CLE1*^*1-420*^ and Mp*CLE1*^*1-417*^). The former contains the 12 amino-acid CLE peptide motif while the latter lacks the C-terminal asparagine residue of the peptide motif, an essential residue for CLE peptide activities [5, 23, 24]. As expected, Mp*CLE1*^*1-420*^ overexpression but not Mp*CLE1*^*1-417*^ overexpression exhibited growth defects (S3 Fig), supporting the notion that the 12 amino-acid CLE peptide is the functional domain of MpCLE1.

### MpCLE1 reduces proliferative activity in the apical notch

**Fig 3:**
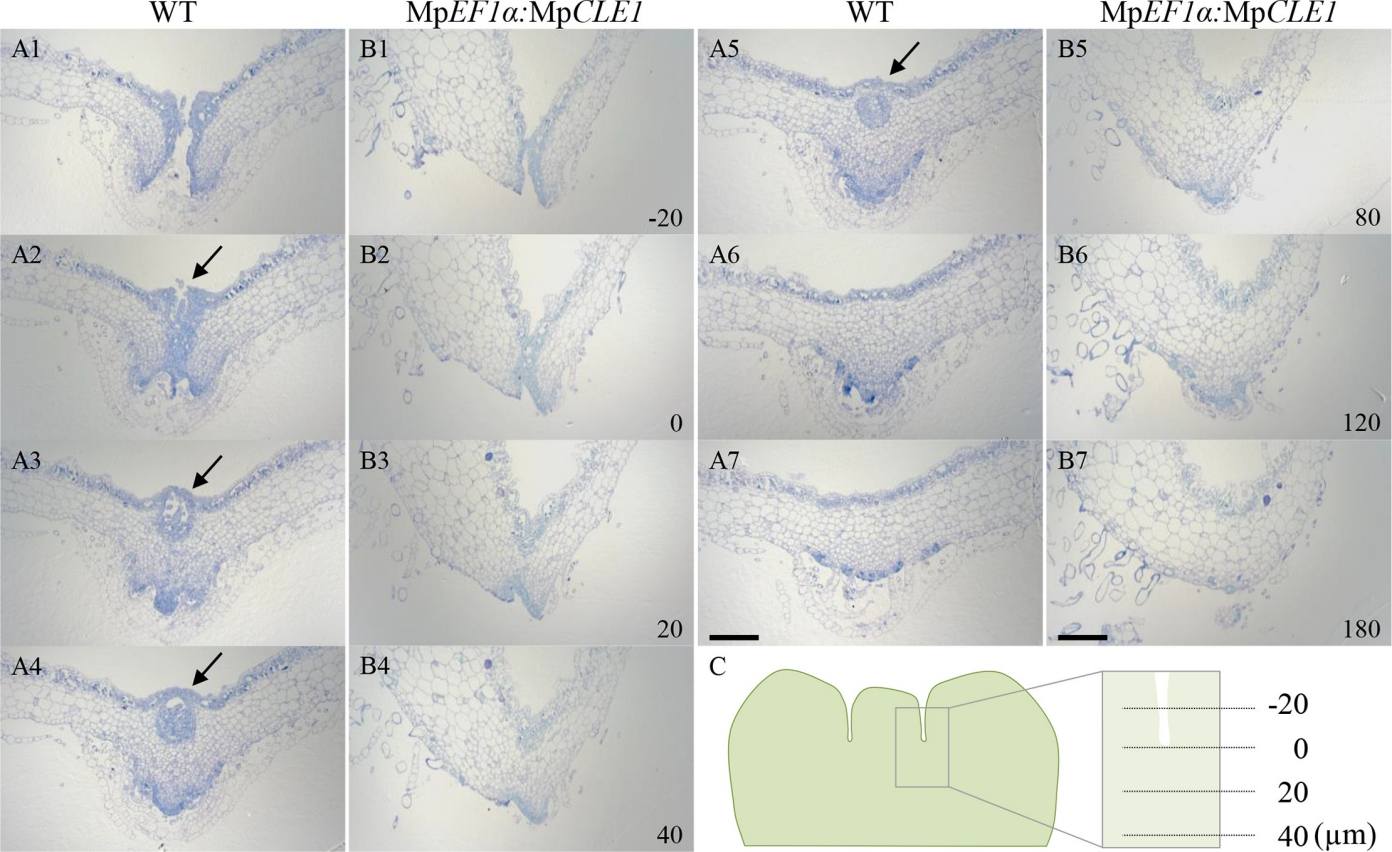
Reduction of meristem activity by Mp*CLE1*. Consecutive transverse sections of apical notches in 14-day-old plants grown from gemmae. WT (A1-A7) and Mp*CLE1* overexpression (B1-B7) plants are compared. The relative position (μm) is indicated at the right bottom corner of each panel. Note that “0 μm” (A2 and B2) is set at the section in which two flanking lobes merged in the consecutive transverse sectioning as illustrated in (C). Arrows indicate a gemmae cup. Scale bars = 200 μm.

To elucidate the cytological function of Mp*CLE1*, we analyzed apical meristem anatomy in 14-day-old plants grown from gemmae. In wild-type meristems, a single apical cell produces derivatives in four planes—dorsal, ventral and two lateral—and each of these primary derivatives undergoes a stereotypical pattern of divisions producing a 'merophyte' [27, 32]. Cell divisions within merophytes produce a pattern of cells in the mature thallus, that when viewed in longitudinal section, appear as rows of cell files emanating from the apical meristem. In longitudinal sections of wild-type plants (Fig 2D), proliferative region, which can be characterized by vertical cell division planes in internal tissues, is approximately 200 μm from the tip of the thallus (bracket in Fig 2E). In contrast, the proliferative region in Mp*CLE1* overexpression plants was reduced to less than 100 μm from the tip (bracket in Fig 2F), with cell differentiation/expansion occurring at a position closer to the apex, resulting in a distortion of thallus growth. We also examined meristem anatomy in transverse sections (Fig 3A–3C). Consecutive transverse sections of wild-type and Mp*CLE1* overexpression plants were compared at same positions relative to a basal position “0 μm” at which the two lobes flanking the meristem merged (Fig 3C). In wild type, small cytoplasmically dense cells are persistently observed in all sections examined (0–180 μm) while Mp*CLE1* overexpression plants initiate cell expansion at the 40 μm position, and larger cells are observed compared to wild type throughout the sections resulting a thickened dorsi-ventral axis (Fig 3A and 3B). Collectively, these data demonstrate Mp*CLE1* overexpression reduces the size of the proliferative region in the apical notch.

### Negative effects of intrinsic Mp*CLE1* on the meristem activity

A loss-of-function Mp*CLE1* allele (Mp*cle1-GT85*) was generated via gene targeting (S4 Fig). Fourteen-day-old Mp*cle1-GT85* plants grown from gemmae formed convoluted thalli, with the thallus periphery curled upward and the apical notches growing downward (epinastically) into the medium. Unlike Mp*CLE1* overexpression plants, overall growth was not reduced (S4 Fig).

**Fig 4:**
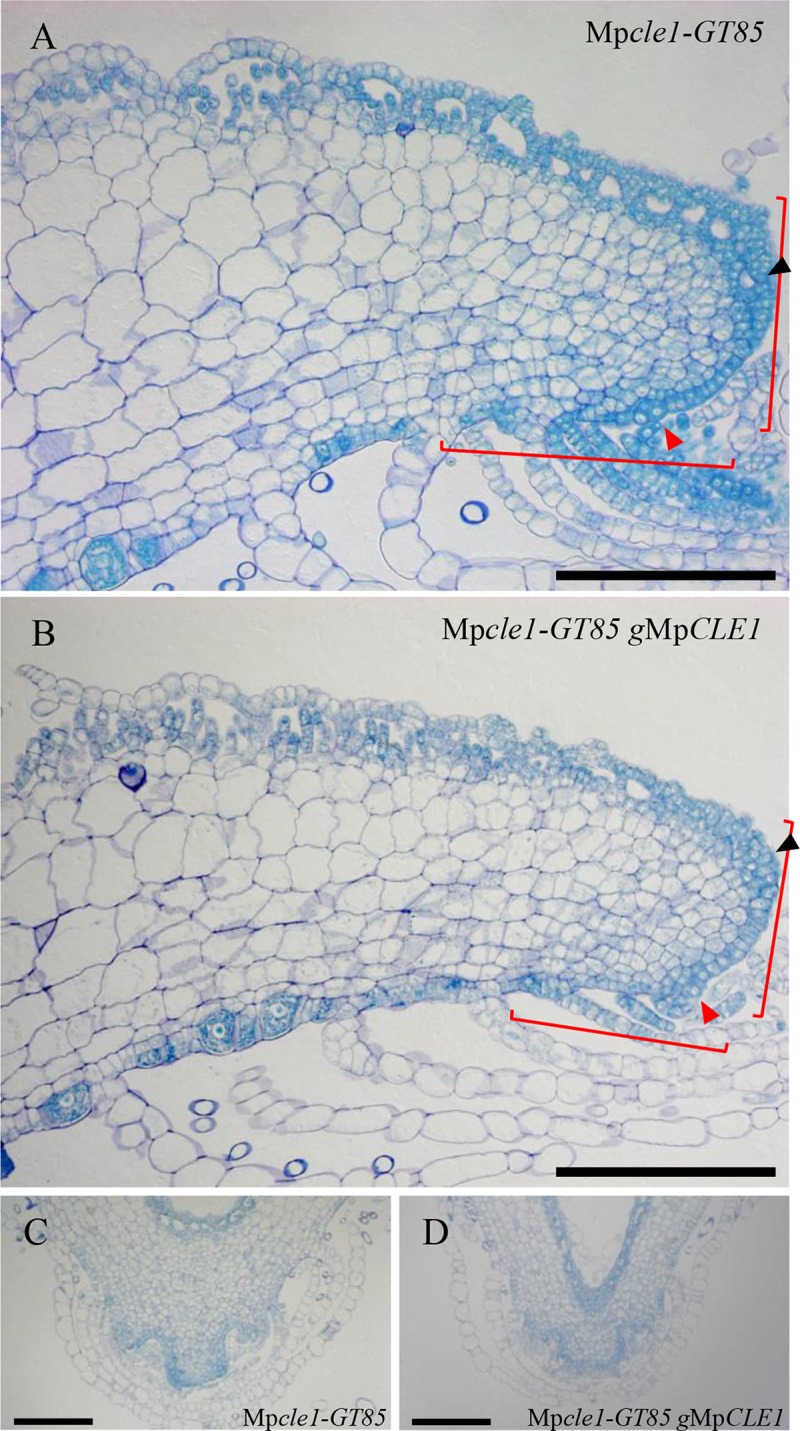
Loss-of-function phenotypes of Mp*CLE1* in apical notch. (A and B) Longitudinal sections of apical notches in 14-day-old *M*. *polymorpha* plants grown from gemmae in (A) homologous recombination-based Mp*cle1* knock-out line and (B) its complementation line. (C and D) Transverse sections of apical notches at “20 μm” position from the junction of two flanking lobes. Note that consecutive sections including these figures are shown in S6 Fig. Red and black arrowheads, and brackets indicate apical cells, developing air chambers closest to the apical cell, and proliferative region of meristem, respectively. Scale bars = 200 μm.

To gain a better understanding of the effects of the loss of Mp*CLE1*, we examined the anatomy of Mp*cle1-GT85* plants. In longitudinal sections of Mp*cle1-GT85* plants, the proliferative region was expanded (n = 2; 266 and 232 μm in length) compared to 200 μm in wild type (bracket in Fig 4A), although the proliferative region was not as clear as in the wild type due to the less organized cell division plane orientation. In contrast, the timing of cell differentiation in epidermal tissue on the dorsal side is not significantly altered in the mutant since the air chamber development started at similar positions from the apical cells (Fig 4A). Complementation analysis was performed by introducing a 6.2 kb genetic fragment spanning Mp*CLE1* into the knock-out line, designated as Mp*cle1-GT85 g*Mp*CLE1*. Compared to the knock-out mutant, the complementation line had a proliferative region similar in size to that of wild type in both internal (n = 2; 198 and 196 μm) and epidermal tissues, but the orientation of cell division planes in internal tissues is still less organized than in wild type (Fig 4B). Curled thalli were also observed in the complementation line (S4 Fig). Thus, additional genomic regulatory components might be required to fully complement the phenotype. In consecutive transverse sections, dorsi-ventral thickening of thalli was observed in Mp*cle1-GT85* plants compared to the complementation line (Fig 4C and 4D). In the consecutive sections, cell expansion continued until the 320 μm position in Mp*cle1-GT85* while it ceased at 200 μm in wild type and the complementation line (S5 Fig). These data suggest that Mp*CLE1* acts to suppress proliferative activity at the apical notch.

**Fig 5:**
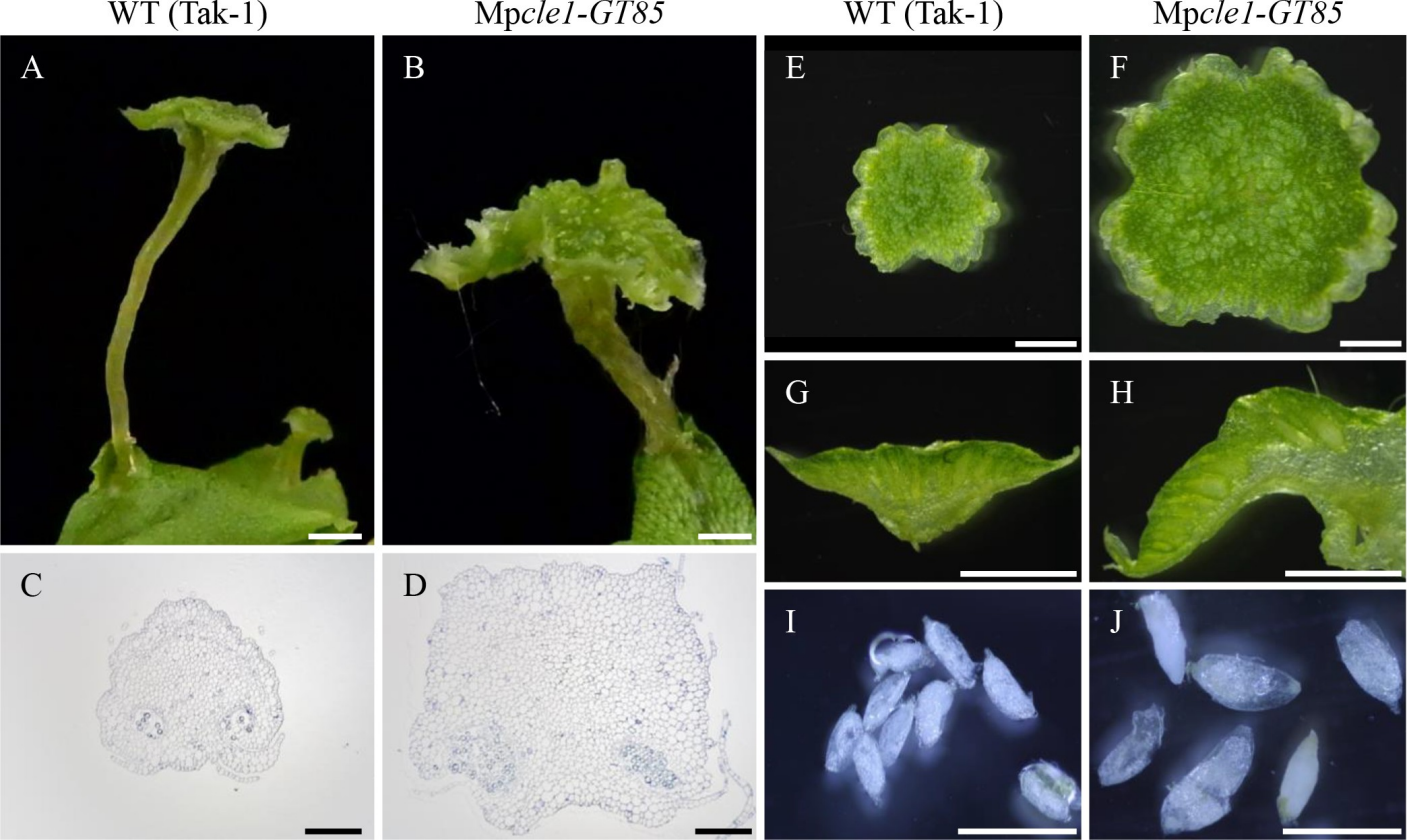
Loss-of-function phenotypes of Mp*CLE1* in antheridiophore. Comparison of antheridiophore morphology between WT (A, C, E, G and I) and Mp*cle1* knock-out line (B, D, F, H and J). (A and B) Overall morphology. (C and D) Stalk cross-sections. (E and F) Antheridial receptacles. (G and H) Hand sections of the antheridial receptacles. (I and J) Antheridia. Scale bars = 2 mm in (A, B and E-H), 1 mm in (I) and (J) and 200 μm in (C) and (D).

**Fig 6:**
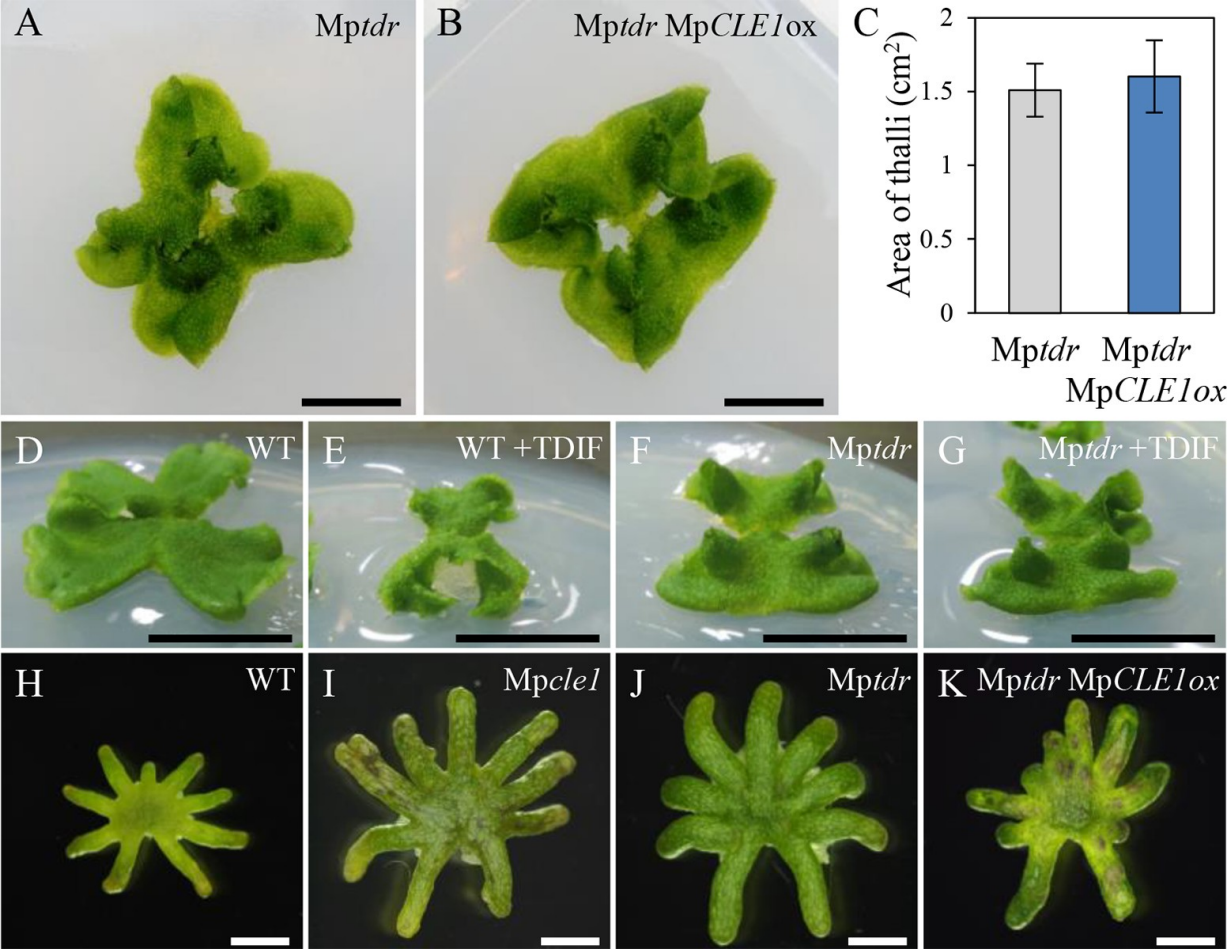
Genetic interaction of Mp*CLE1* and Mp*TDR*. Mp*tdr* knock-out mutant is insensitive to excess MpCLE1/TDIF. (A and B) Overall morphology of 21-day-old plants grown from gemmae. (C) Area of thalli in 14-day-old plants (mean ± s.d.). No significant difference was found between Mp*tdr* and Mp*tdr* Mp*CLE1ox* in Weltch’s *t*-test (p = 0.19, n = 17–18). (D-G) Overall morphology of 17-day-old plants grown from gemmae with or without 10 μM TDIF. (H-K) Archegonial receptacles. Mp*cle1* and Mp*tdr* indicate knock-out mutants. Mp*CLE1ox* indicates Mp*EF1α* promoter-driven overexpression. Note that Mp*CLE1* overexpression plants do not make gametangiophores (S3G Fig). Scale bars = 1 cm in (A,B,D-G) and 2 mm in (H-K).

Gametangiophores, specialized reproductive structures, are extensions of the vegetative thalloid body and the proliferative activity of apical meristem. Mp*cle1-GT85* plants developed larger antherodiophores (male gametangiophores) than wild type (Fig 5A and 5B). Consistent with the increased proliferation in vegetative thalli, stalks are thicker in Mp*cle1-GT85* plants compared to wild type (1.12 ± 0.14 mm v.s. 0.65 ± 0.14 mm, mean ± S.D., n = 10) and are composed of more cells in cross sections (Fig 5C and 5D). The diameter of the antheridial receptacle was also increased in both longitudinal and transverse axes in Mp*cle1-GT85* (Fig 5E and 5F, S6 Fig). The mutant receptacles were thicker and contained larger antheridia, which can reach 1.12 mm in length at the maxima (mean ± S.D. = 0.80 ± 0.14 mm, n = 28) in contrast to wild-type antheridia of 0.70 mm in length at the maxima (mean ± S.D. = 0.55 ± 0.08 mm, n = 17) in our observation (Fig 5G–5J), which is consistent to Higo et al. [33]. The complementation line developed normal antheridiophores and antheridia of 0.66 mm in length at the maxima (mean ± S.D. = 0.56 ± 0.08 mm, n = 19) (S4 Fig). Gametangiophore overgrowth was also consistently observed in archegoniophores of female Mp*cle1-GT85* plants obtained by cross with Tak-2, without affecting the branching pattern of fingered-lobes (Fig 6H and 6I). These observations support the notion that Mp*CLE1* negatively controls the proliferative activity in apical meristems.

### Genetic interaction of Mp*CLE1* and Mp*TDR*

To examine the ligand-receptor relationship between TDIF and TDR in *M*. *polymorpha*, we analyzed the physiological function of the *TDR* ortholog, Mp*TDR*, by generating a knock-out line via homologous recombination (S4 Fig; Mp*tdr-GT400*). Similar to Mp*cle1-GT85*, 21-day-old Mp*tdr-GT400* thalli were curled upward at the periphery (Fig 6A). In this mutant background, introduction of an Mp*CLE1* overexpression transgene did not alter thallus morphology (Fig 6B). The area of thalli was not significantly changed between Mp*tdr-GT400* and Mp*CLE1* overexpression in Mp*tdr-GT400* background (Fig 6C), indicating that Mp*CLE1* activity is dependent on Mp*TDR*. Consistently, development of Mp*tdr-GT400* thalli was insensitive to 10 μM TDIF, the most effective MpCLE1-type peptide in our assays (Fig 6D–6G). The archegoniophores of Mp*tdr-GT400* plants were larger than wild type, resembling the female Mp*cle1-GT85* phenotype (Fig 6H–6J). Introduction of Mp*CLE1* overexpression did not significantly alter the archegoniophore morphology of Mp*tdr-GT400* (Fig 6K). Collectively, these data indicate that Mp*CLE1* acts through Mp*TDR* to restrict the proliferative activity in the meristems of *M*. *polymorpha*.

### Expression of Mp*CLE1* and Mp*TDR* in apical meristem

**Fig 7:**
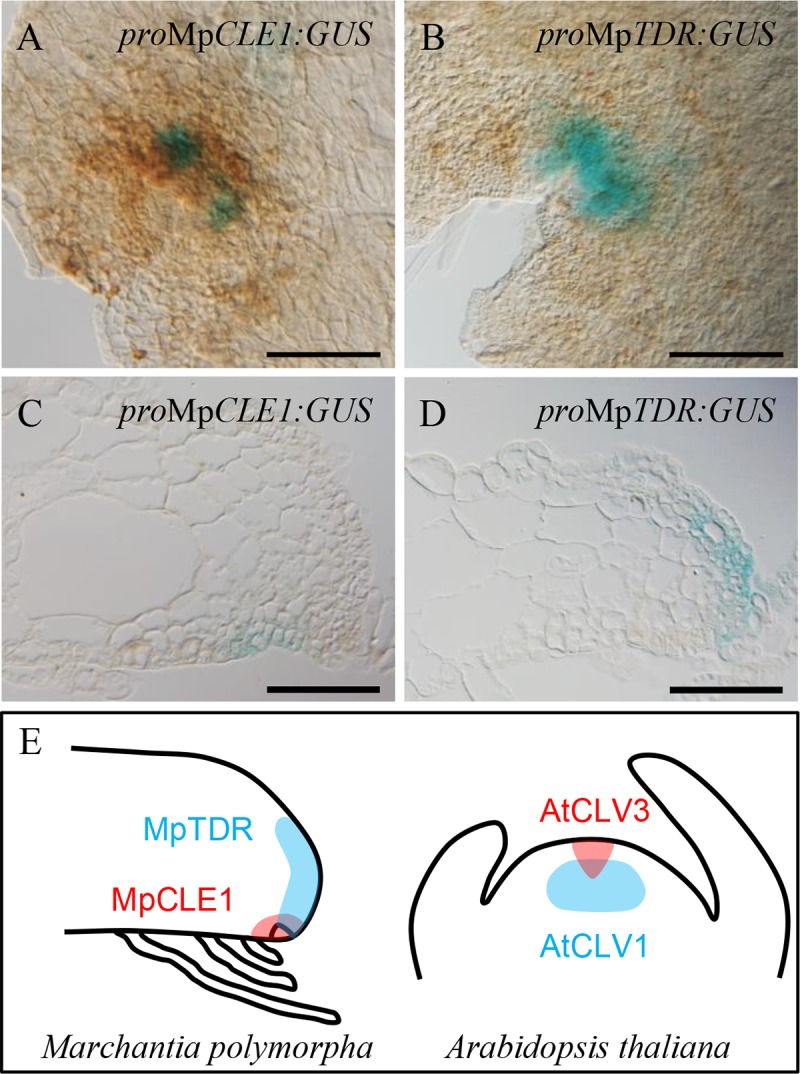
Expression patterns of Mp*CLE1* and Mp*TDR* in apical notch. (A and B) Promoter-GUS reporter assays for Mp*CLE1* and Mp*TDR* expression in 5-day-old gemmalings. (C and D) Longitudinal sections of the promoter:GUS lines at the notch in 5-day-old gemmalings. (E) Schematic illustration of promoter activities of CLE (red) and receptor (cyan) genes in the meristem. *M*. *polymorpha* (left) genes are contrasted with *A*. *thaliana* (right) genes. Scale bars = 200 μm (A and B), 100 μm (C and D).

To analyze the expression patterns of Mp*CLE1* and Mp*TDR*, we constructed GUS-reporter lines using 5 kb of genomic sequence upstream of the Mp*CLE1* and Mp*TDR* coding sequences, respectively. GUS signal for both MpCLE1 and MpTDR promoters was first detected in the apical notches in 5-day-old gemmalings (Fig 7A and 7B). In 10-day-old gemmalings, _*pro*_Mp*CLE1*:*GUS* signal was also detected along the midrib in addition to the signals at the apical notches (S7 Fig). Both _*pro*_Mp*CLE1*:*GUS* and _*pro*_Mp*TDR*:*GUS* signals were observed in the developing antheridiophores suggesting that MpCLE1 signaling is functional during the development of antheridiophores (S7 Fig). In longitudinal sections, _*pro*_Mp*CLE1*:*GUS* signal was detected in a small area around the apical cell in 5-day-old gemmalings (Fig 7C). In contrast, _*pro*_Mp*TDR*:*GUS* signal was detected in the dorsal part in the apical meristem (Fig 7D), suggesting that MpCLE1 signaling may operate within the apical meristem. Since the expression of _*pro*_Mp*TDR*:*GUS* was observed in cells close to the apical cell, the expression domains of Mp*CLE1* and Mp*TDR* could partially overlap. The influence of MpCLE1 signaling on the expression domains was examined by peptide treatment. In 5-day-old gemmalings grown in the presence of 10 μM MpCLE1-Hyp or TDIF peptide, _*pro*_Mp*CLE1*:*GUS* signals were not conspicuously affected, while _*pro*_Mp*TDR*:*GUS* signals appeared more intense than in the control, however, no change in expression levels was detected in fluorometric quantification assays (S7 Fig). In whole-mount *in situ* hybridization (WISH) assays, Mp*CLE1* expression was detected at the apical notches of 7-day-old gemmalings (S8 Fig). MpEF1α-as signal was detected throughout the thallus and strongly in apical notches while MpEF1α-s was not, consistent with Althoff et al. [34] (S8 Fig). Collectively, these data suggest that the MpCLE1 peptide signal may move from a ventral region towards more dorsal regions, with a maximum potential response, i.e. Mp*TDR* expression, near the apical cell.

## Discussion

In this work, we show that TDIF-type CLE peptide signaling restricts proliferative activity in the meristem and the overall size of reproductive organs in the liverwort *M*. *polymorpha* although development of apical cell, merophyte, or cell division markers such as labile cyclin-GUS is important to unambiguously quantify the change of proliferative activity and its location in the meristem [35]. TDIF has been known as a positive regulator of meristem activity in vascular plants, but conversely MpCLE1/TDIF acts as a negative regulator in *M*. *polymorpha*. This function resembles CLV3 peptides in flowering plants. In Arabidopsis *clv3* mutants, the shoot and floral meristems are enlarged, resulting in club-shaped siliques and additional floral organs [36]. Tomato mutants and cultivars deficient in CLV3 signaling have excess floral organs and fasciated fruits [37]. In monocots, loss of CLV signaling enhances the meristem activity, which results in the increase of floral organ number in rice and kernel row number in maize [38–43]. Since the reproductive structures are determinate in these plants, the effects of enhanced proliferative activities are more conspicuous than effects on vegetative growth. Correlation of proliferative activity in the meristem and reproductive structure size was also found in Mp*cle1* mutant that exhibits gametangiophore enlargement along with the enhanced proliferative activity. In contrast to the phenotypes in flowering plants, the gametangiophores of the *M*. *polymorpha* mutants did not form an excess number of reproductive structures, such as additional lobes in the receptacles or fasciated stalks. The lobes and stalks are both sexual extensions of thallus and the numbers of these structures depend on the branching of the meristems during gametangiophore development. Thus, the control of proliferative activity by MpCLE1 signaling is uncoupled from meristem branching. In addition to the functional similarity, the expression pattern of Mp*CLE1* relative to its receptor gene, Mp*TDR*, also resembles to that of *CLV3* relative to *CLV1* in Arabidopsis, in which the peptide ligand is expressed at/around stem cells while the receptor is expressed in a neighboring region (Fig 7E) [44,45]. In Arabidopsis, receptor expression in the partially overlapping domain with the ligand is thought to establish different cell fates among neighboring target cells [11, 12, 46]. In *M*. *polymorpha*, proliferative cells in the dorsal region of apical meristem could also interpret MpCLE1 signals from apical cells as a positional cue. However, the morphological phenotypes of knock-out and overexpression mutants for Mp*CLE1* were found more significantly in the internal tissue. Further identification of downstream signaling components that suppress proliferative activity would clarify the role of MpCLE1-mediated communication in meristem homeostasis.

The slow but continued thallus growth in Mp*CLE1* overexpression lines indicates that ectopic MpCLE1 does not terminate stem cell activity, and indeed apical cells are maintained in overexpression lines. This contrasts with Arabidopsis CLV3 overexpression, which terminates shoot growth by abolishing stem cells in the meristem [12, 13] and with Arabidopsis TDIF mutants that lose vascular stem cells at a certain frequency [47]. Loss of Mp*CLE1* also resulted in abnormal cell division planes in the meristem, similar to both loss and ectopic expression of TDIF in Arabidopsis [21, 22]. Therefore, the cellular function of Mp*CLE1* signaling appears to be a combination of CLV3 and TDIF signaling observed in Arabidopsis. Likewise, it was recently demonstrated that CLV3 orthologs in *P*. *patens* also regulate both cell proliferation and cell division orientation planes in gametophore shoots and that this function is conserved in Arabidopsis [48]. Coupled with our results, these observations hint that perhaps the ancestral functions of both the TDIF and CLV3 pathways may have encompassed regulation of both cell proliferation and cell division orientation planes.

Since TDIF and TDR are genetically conserved in vascular plants, lack of TDIF and TDR in the moss *P*. *patens* suggests that TDIF signaling has been lost within the moss lineage. Thus, TDIF signaling is not an indispensable regulatory module for *P*. *patens* development despite its significant contribution to *M*. *polymorpha* development, perhaps due to the major differences in body plan between mosses and liverworts [49]. In contrast, *M*. *polymorpha* retains both TDIF (Mp*CLE1*) and CLV3 (Mp*CLE2*) orthologs suggesting each possesses unique functions in liverworts.

Our work reveals a general association of CLE peptide signaling with both gametophytic and sporophytic meristems in land plants although it’s still unclear if CLE is involved in the control of sporophytic meristems in bryophytes. Meristems in both generations evolved in the ancestral land plant and are not present in algae which do not possess CLE peptide signaling. One major difference in the body plans of these organisms is that the land plant body is built from meristems with 3 or more cutting faces, whereas algal bodies are largely constructed from modifications of filamentous growth so that three-dimensional co-ordination of cell behavior is not required. Thus, mechanisms to focus and control meristem growth evolved concomitantly with meristems, with one mechanism being via CLE peptide-based cell-to-cell communication.

## Materials and methods

### Phylogenetic analysis

Phylogenetic analysis was performed as described previously [25]. Sequences for phylogenetic analysis were summarized in S1 Table. The sequences were first aligned with Clustal X and Bayesian phylogenetic analyses were performed on the alignments using MrBayes 3.2.1 [50].

### Plant materials and growth conditions

Columbia-0 (Col-0) line of *Arabidopsis thaliana* and Takaragaike-1 (Tak-1) and BC3-38 lines of *M*. *polymorpha* were used as wild type in phenotypic analyses and as the genetic background for transgenic lines. Growth conditions for Arabidopsis and observation methods of vasculature were as described previously [14]. *M*. *polymorpha* plants were grown at 22°C on half-strength Gamborg B5 medium (pH 5.5, 1.4% agar) under continuous light. For the induction of gametangiophores, far-red light was supplemented. For the observation of antheridia, antheridial receptacles were dissected with forceps under a stereoscopic microscope (Stemi 2000-CS, Zeiss, Jena, Germany). To measure the area of thalli, gemmae were grown on the half-strength Gamborg B5 medium for 14 days and images of plants were analyzed using ImageJ.

### Peptide synthesis

Peptides were synthesized by Fmoc chemistry with a peptide synthesizer (CS136XT, CSBio, CA, USA). Analytically pure peptides were obtained by reverse-phase HPLC.

### Construction and transformation

Primers, plasmids and transgenic plants are summarized in S2 Table and S3 Table [51–54]. Transformation of *M*. *polymorpha* was performed using spores (cross between Tak-1 and Tak-2) or regenerating thalli according to Ishizaki et al. [51] and Kubota et al. [52]. Homologous recombination-mediated gene targeting was performed according to Ishizaki et al. [53]. Transformation of Arabidopsis was performed with the floral dip method [55].

### Quantitative RT-PCR (qRT-PCR)

Total RNA was extracted from 11-day-old Arabidopsis seedlings with RNeasy plant mini kit (Qiagen, Hilden, Germany). Three independent RNA samples were used for cDNA synthesis with SuperScript III first-strand synthesis system (Thermo Fisher Scientific, MA, USA). Two technical replicates are made for each RNA sample and the average was used as a single data point. Primers used for qPCR are described in S2 Table. The qPCR assay was performed on a LightCycler 96 system (Roche) using KAPA SYBR FAST qPCR kit (KAPA BIOSYSTEMS, MA, USA). Amounts of cDNA input to the qPCR reactions were normalized by the At*TUA4* expression levels. For the comparison of At*CLE41* and Mp*CLE1* gene expression levels, the expression levels were normalized by performing qPCR using 10 pg of cloning plasmid (pENTR-AtCLE41 or pENTR-MpCLE1) as template. Mean values of 3 samples ± S.D. were indicated.

### GUS reporter assay

GUS staining was performed according to Ishizaki et al. [51]. Briefly, *M*. *polymorpha* gemmalings grown on agar medium were directly submerged in X-Gluc assay solution containing 50mM sodium phosphate buffer (pH 7.2), 1mM potassium-ferrocyanide, 1mM potassium-ferricyanide, 10mM EDTA, 0.01% Triton X-100 and 1mM 5-bromo-4-chloro-3-indolyl-β-D-glucuronic acid. After vacuum infiltration, samples were incubated for 3–12 hours at 37°C in dark. GUS-stained samples were cleared with ethanol and mounted with clearing solution (chloral hydrate-glycerol-water, 8:1:2) before imaging with light microscope (Axio Imager.A2, Zeiss). For histological analyses, GUS-stained samples were rinsed with water before fixation. At least 2 independent transgenic lines were examined for each experiment and representative images are shown. Fluorometric quantification of GUS activity was performed according to Ishizaki et al. [56] with minor modifications. Three biological replicates were sampled. For each replicate, 5 gemmalings (5–10 mg in total) grown for 5 days on the half-strength Gamborg B5 medium with or without 10 μM peptide were collected in a microtube, frozen with liquid nitrogen and homogenized with micropestle in 100 uL of GUS extraction buffer, containing 50 mM sodium phosphate (pH 7.2), 10 mM 2-mercaptothanol, 1 mM EDTA and 0.01% Triton X-100. Debris was removed by centrifugation at 13,000 rpm for 5 min at 4°C. Protein concentration was measured using 5 uL of the protein solution using the TaKaRa Bradford Protein Assay Kit (Takara, Kyoto, Japan) with the low-concentration protocol according to the manufacturer’s instructions. For the GUS enzyme reaction, 40 μl of each protein solution (ca 30 μg protein), 50 μl of the GUS extraction buffer and 10 μl of 10 mM 4-methylumbelliferyl β-D-glucuronide (MUG) was mixed in a microtube and incubated at 37°C for 40 min. The reaction was stopped by adding 900 μl of 200 mM sodium carbonate. Fluorescence (460 nm emission/360 nm excitation) of liberated 4-methylumbelliferone (MU) was measured on a microplate reader (Synergy LX, BioTek, VT, USA) and normalized by the protein concentration.

### Histology

For preparation of plastic sections, plant samples were trimmed with a razor blade and fixed in FAA solution (50% ethanol: 10% formalin: 5% acetic acid in water). Fixed samples were embedded into Technovit 7100 resin (Heraeus Kulzer, Wehrheim, Germany) and 4 μm sections were prepared with a rotary microtome (RM2235, Leica, Heidelberg, Germany). Sections were stained with 0.02% toluidine blue or with 0.002% Safranin-O solution for GUS-stained samples and then mounted with Entellan New (Merck Millipore, MA, USA).

### Whole mount *in situ* hybridization (WISH)

WISH was performed by modifying a protocol for Arabidopsis seedlings [57]. For the preparation of RNA probes, Mp*CLE1* and Mp*EF1α* genes were cloned into pCRII vector (Thermo Fisher) by PCR from *M*. *polymorpha* cDNA using primers indicated in S2 Table. Digoxygenin-labeled ribo-probes were synthesized using SP6/T7 RNA polymerases (DIG RNA Labeling Kit, Roche, Basel, Switzerland) after digestion with *Xho* I/*Bam*H I restriction enzymes, respectively. For the preparation of plant samples, 10-day-old gemmalings were fixed in a 1:1 mixture of heptane and fixative (4% paraformaldehyde (PFA), 15% DMSO and 0.1% Tween- 20 in water) for 45 min on a rotary shaker at room temperature (RT). Following fixation, tissues were placed in 100% methanol twice for 5 min and 100% ethanol three times for 5 min to remove chlorophyll and incubated for 30 min in a 1:1 mixture of ethanol and Histo-Clear. After treatment of 100% ethanol, tissues were rehydrated in 75% ethanol (v/v in water), 50% ethanol (v/v in phosphate buffered saline; PBS) and 25% ethanol (v/v in PBS) for 10 min each. Tissues were refixed in the fixative for 20 min and rinsed twice for 10 min in PBST (0.1% v/v Tween-20 in PBS) at RT. For permeabilization of cell wall, tissues were treated with 0.1% cellulase (final concentration 100 μg/ml) and 0.2% macerozyme (final concentration 200 μg/ml) for 30 min at RT and with proteinase K (final concentration 125 μg/ml) for 30 min at RT. After stopping the permeabilization with glycine (final concentration 2mg/ml), tissues were refixed in the fixative and rinsed in PBST for 10 min at RT. For labeling, tissues were hybridized with the DIG-probes (150 μg/ml) in the hybridization solution (50% formamide in 5×saline-sodium citrate buffer (SSC) containing 0.1% Tween-20, 0.1 mg/ml heparin and 0.1mg/ml herring sperm DNA) for 16 hours at 55°C. The probe mixture was denatured prior to use for 10 min at 80°C. Hybridized tissues were rinsed in 4×SSC three times for 15 min at 55°C, 0.1×SSC three times for 20 min at 55°C and maleic acid buffer (MAB) for 5 min at RT. After washing excessive probe, tissues were incubated in 0.1% boheringer blocking reagent in maleic acid buffer (BBR-MAB) for 30 min at RT, the 1:2,000 diluted anti-digoxigenin-AP Fab fragments (Roche) in BBR-MAB for 2 hours at RT and 0.05% (v/v) Tween-20 in MAB three times for 15 min at RT. Bound ribo-probe was detected by overnight staining with nitroblue tetrazolium (Roche) and bromo-chloro-indolyl phosphate (Roche) for overnight at 4°C. Stained tissues were photographed under a stereoscopic microscope (MZ16F, Leica).

## Supporting information

S1 Fig CLE proteins in *M. polymorpha*. Deduced protein sequences of MpCLE1 and MpCLE2. Signal peptides are underlined and the 12 amino-acid CLE peptide motifs are highlighted in red.
(TIF)

S2 Fig MpCLE1 does not act as TDIF in Arabidopsis. Effects of MpCLE1 was examined in Arabidopsis plants. (A-C) Xylem formation in 14-day-old leaves. (D-F) Vascular development in 10-day-old hypocotyls. (G-I) Overall morphology of 4-week-old plants. (J) Relative expression levels of At*CLE41*/Mp*CLE1* genes in 11-day-old plants. Expression levels of two CLE genes were normalized by absolute quantification with external references (cloning plasmid). (K-O) Xylem formation in 14-day-old leaves grown in liquid medium with or without peptides. (P-R) Vascular development in hypocotyls in plants grown in liquid medium with or without peptides for 10 days. Red and cyan lines indicate vein and xylem strand, respectively in (A-C) and (K-O). Red bars in (D-F) and (P-R) indicate stele widths. Scale bars = 100 μm in (A-F, K-R) and 2 cm in (G-I).
(TIF)

S3 Fig MpCLE1 affects thallus development in *M. polymorpha*. (A-F, H and I) Overall morphology of 14-day-old plants grown from gemmae. Pictures taken at oblique angles are also indicated in (A-F) to show the convolution of thalli. (G) Mp*CLE1* overexpression plants grown for 2 months under far red-supplemented light for reproductive induction failed to produce gametangiophores. Scale bars = 5 mm.
(TIF)

S4 Fig Verification of homologous recombination lines. (A) Genotyping scheme for gene targeting by homologous recombination (left). WT, HR and RI indicate wild-type, homologous recombination and random insertion genotypes, respectively. In the screening experiments for HR line, many RI lines are generated and primers, c and d, are used to distinguish HR from RI. Genotyping for Mp*cle1* and Mp*tdr* gene-targeting mutants (right). Three different primer sets (a-b, c-x, d-y) for each gene were used in genomic PCR to detect wild-type specific (yellow arrows) and mutant-specific (cyan arrows) products. Lanes (wt and mut) are for the PCR products from wild type (Tak-1) and mutant (Mp*cle1-GT85* or Mp*tdr-GT400*) DNA samples, respectively. (B) 14-day-old plants grown from gemmae for complementation test. (C) The antheridiophore morphology of Mp*CLE1* complementation line. See also Fig 4.
(TIF)

S5 Fig Increase of meristem activity in Mp*CLE1* mutant. Consecutive transverse sections of apical notches in 14-day-old plants grown from gemmae. Comparison of WT (A1-A7), Mp*cle1-GT85* (B1-B7) and a complementation line (C1-C7), with measurements as in Fig 3. The relative position (μm) is indicated at the right bottom corner of each panel. Scale bars = 200 μm.
(TIF)

S6 Fig Quantification of receptacle size. Diameters of receptacles in two axes (longitudinal and transverse as in right panel) are compared between Tak-1 (wild type) and Mp*cle1-GT85* mutant. Data represents mean values ± s.d. (p<0.05 in Weltch’s *t*-test, n = 10–12).
(TIF)

S7 Fig Analysis of Mp*CLE1* and Mp*TDR* promoter:GUS lines. (A) Promoter-GUS assay for Mp*CLE1* in 10-day-old plants grown from gemmae. Whole mount sample (left) and longitudinal section (right) are shown. Note that GUS signals are detected within the meristem and along midrib. Scale bars indicate 500 μm (left) and 200 μm (right). (B) GUS activities in immature antheridiophores. Scale bars indicate 5 mm. (C) GUS activities in the apical notches of 5-day-old gemmalings grown with or without peptide as indicated above. Scale bars indicate 200 μm. (D) Fluorometric quantification of GUS activities on plants grown in the same conditions as (C). Data represent the mean values ± s.d. of 3 biological replicates. No significant differences were detected between peptide treatment and mock treatment for either *pro*Mp*CLE1*:*GUS* or *pro*Mp*TDR*:*GUS* (p>0.2 in Weltch’s *t*-test).
(TIF)

S8 Fig Whole mount *in situ* hybridization analysis on Mp*CLE1*. Whole mount *in situ* hybridization (WISH) assays in *M*. *polymorpha* thalli with probes as follows: MpCLE1-antisense in (A), MpEF1α-antisense in (B) and MpEF1α-sense in (C). Note that the MpEF1α probes were used for positive and negative controls. Scale bars = 500 μm.
(TIF)

S1 Table Gene sequences. (XLSX)

S2 Table Primers used in this study. (XLSX)

S3 Table Plasmids and transgenic plants made in this study. (XLSX)
