# Supplementary figures and images for: Control of proliferation in the haploid meristem by CLE peptide signaling in Marchantia polymorpha
Source: PLoS Genet. 2019 Mar 7;15(3):e1007997. doi: 10.1371/journal.pgen.1007997 (PMC6424463; doi:10.1371/journal.pgen.1007997)

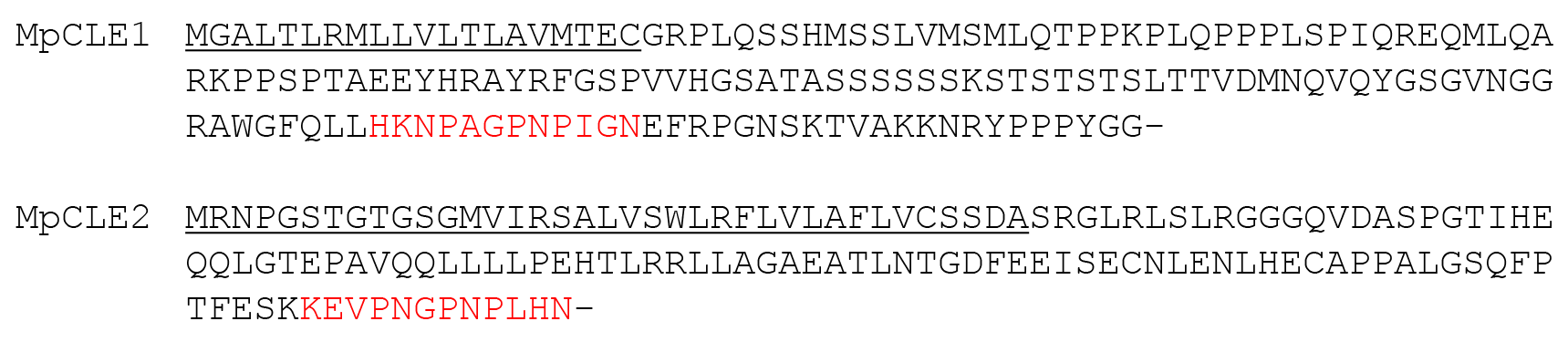

Supplement: S1 Fig — Deduced protein sequences of MpCLE1 and MpCLE2. Signal peptides are underlined and the 12 amino-acid CLE peptide motifs are highlighted in red. (TIF) [file pgen.1007997.s001.tif]

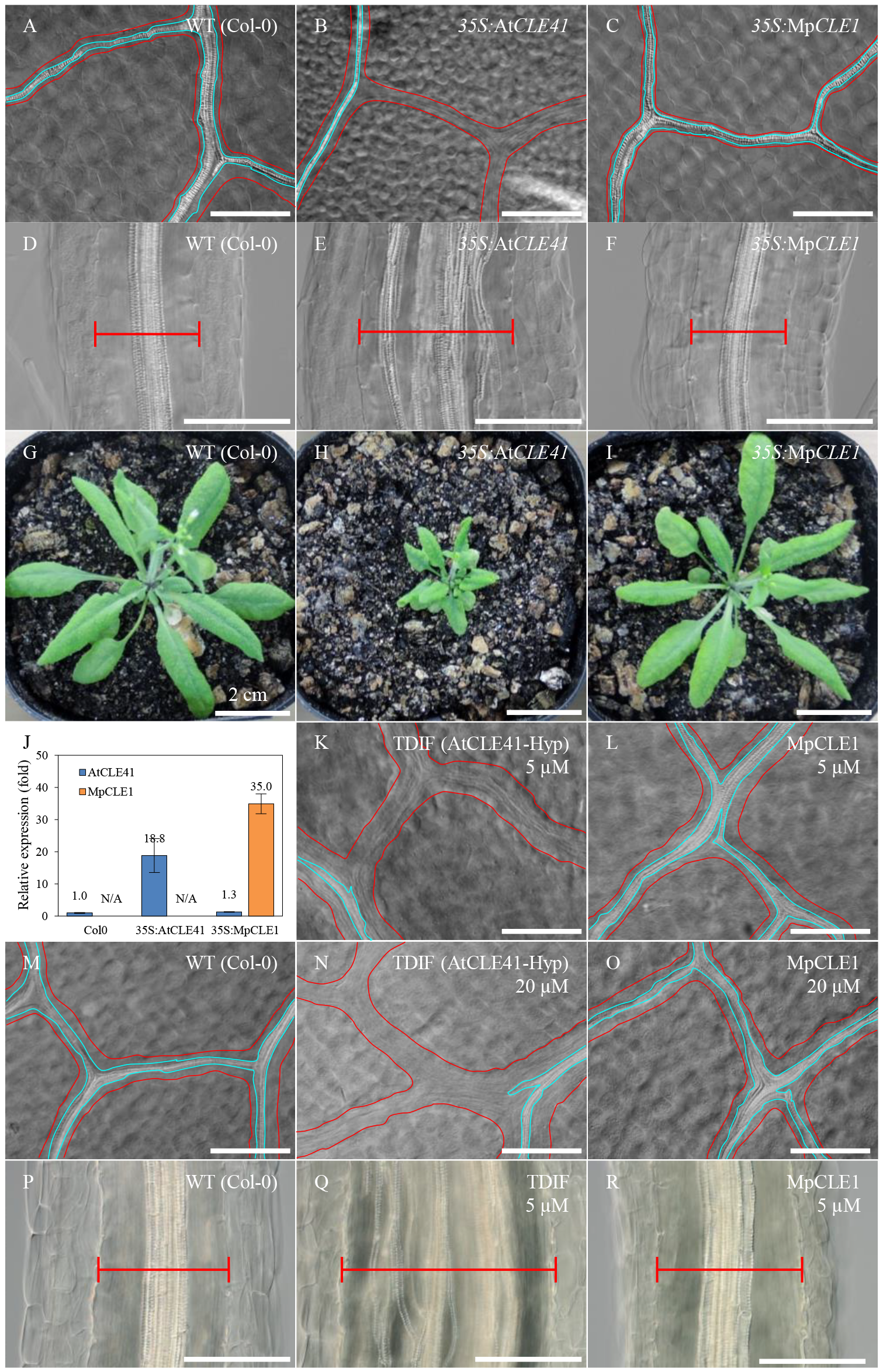

Supplement: S2 Fig — Effects of MpCLE1 was examined in Arabidopsis plants. (A-C) Xylem formation in 14-day-old leaves. (D-F) Vascular development in 10-day-old hypocotyls. (G-I) Overall morphology of 4-week-old plants. (J) Relative expression levels of AtCLE41/MpCLE1 genes in 11-day-old plants. Expression levels of two CLE genes were normalized by absolute quantification with external references (cloning plasmid). (K-O) Xylem formation in 14-day-old leaves grown in liquid medium with or without peptides. (P-R) Vascular development in hypocotyls in plants grown in liquid medium with or without peptides for 10 days. Red and cyan lines indicate vein and xylem strand, respectively in (A-C) and (K-O). Red bars in (D-F) and (P-R) indicate stele widths. Scale bars = 100 μm in (A-F, K-R) and 2 cm in (G-I). (TIF) [file pgen.1007997.s002.tif]

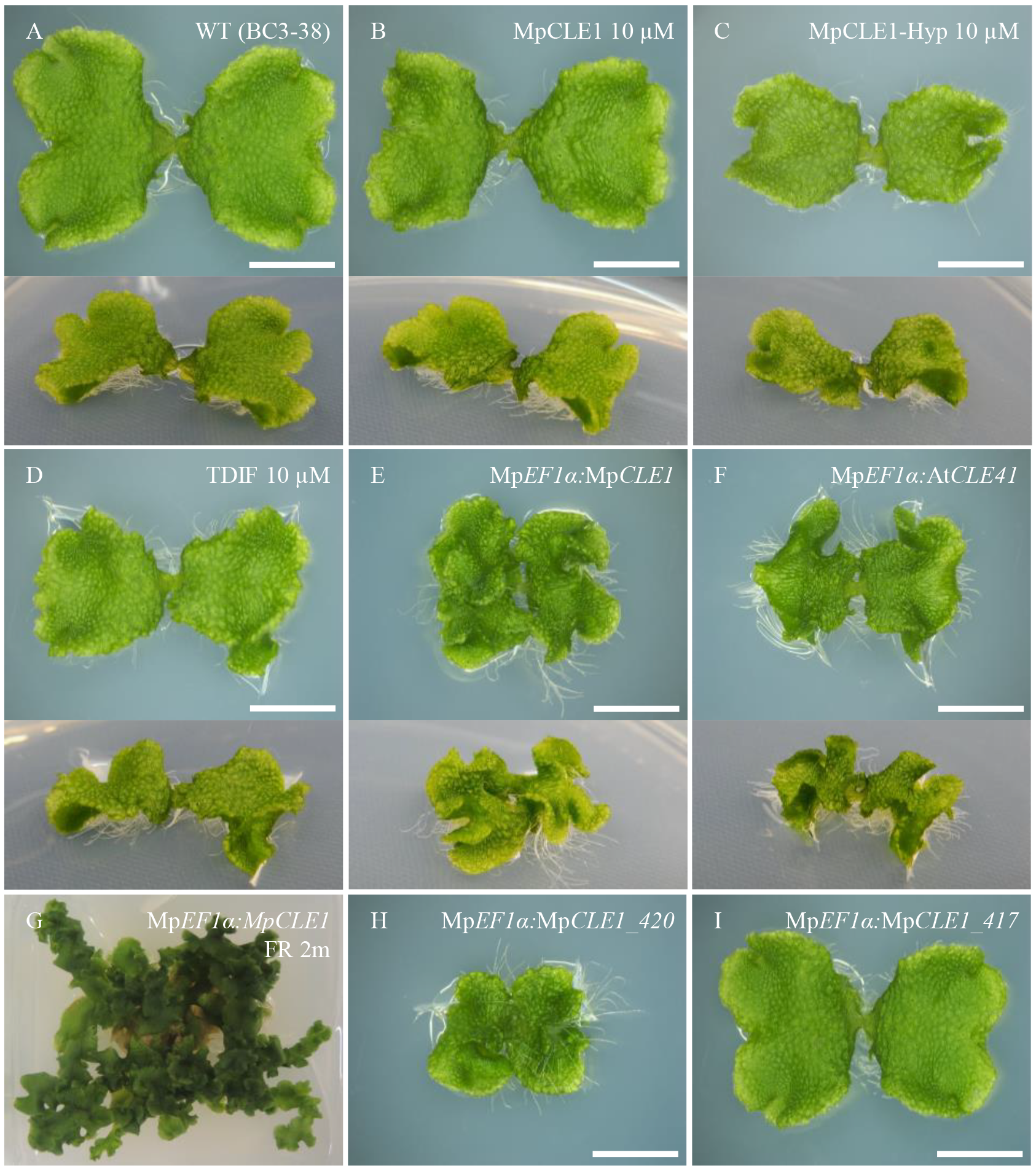

Supplement: S3 Fig — (A-F, H and I) Overall morphology of 14-day-old plants grown from gemmae. Pictures taken at oblique angles are also indicated in (A-F) to show the convolution of thalli. (G) MpCLE1 overexpression plants grown for 2 months under far red-supplemented light for reproductive induction failed to produce gametangiophores. Scale bars = 5 mm. (TIF) [file pgen.1007997.s003.tif]

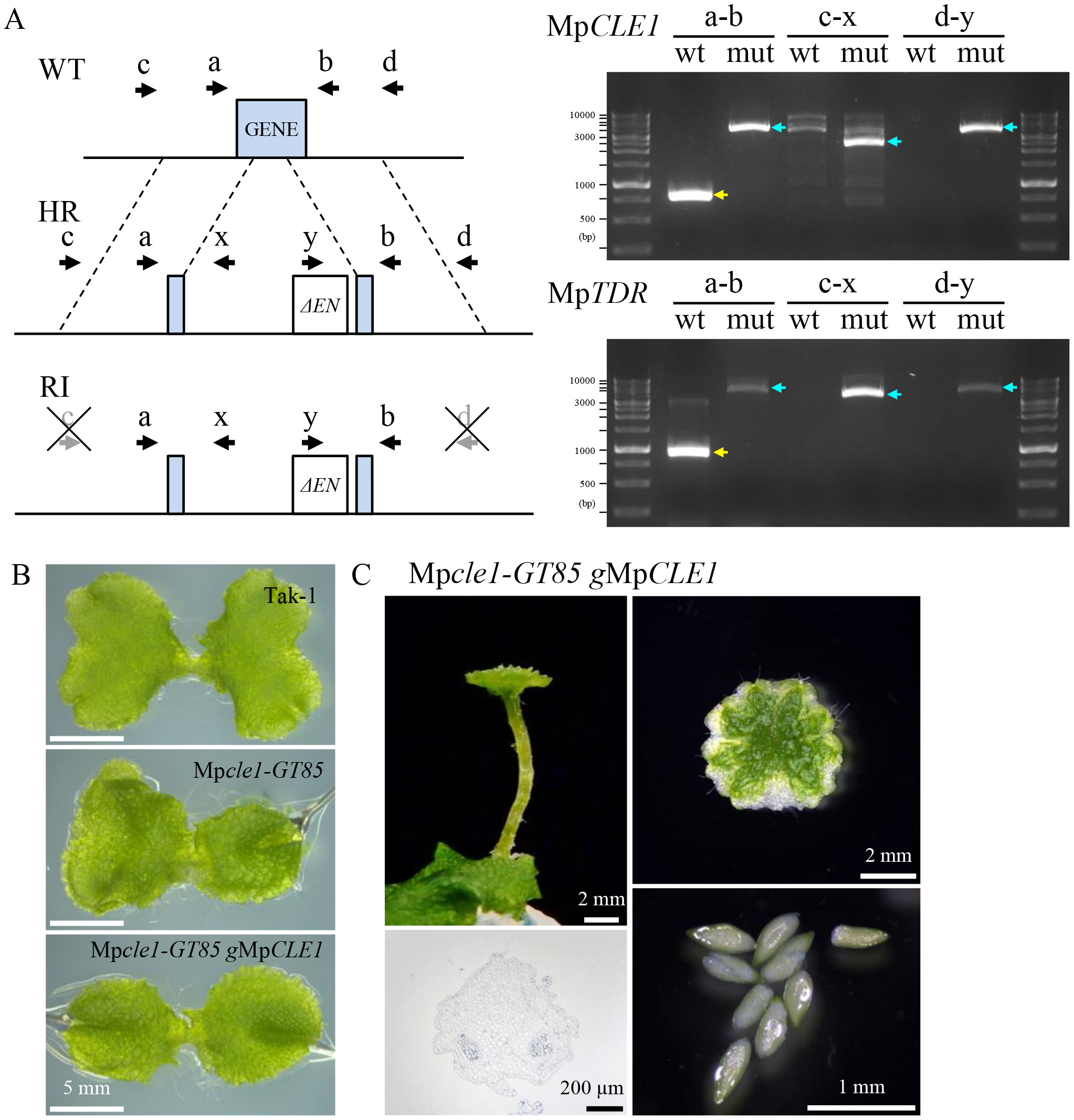

Supplement: S4 Fig — (A) Genotyping scheme for gene targeting by homologous recombination (left). WT, HR and RI indicate wild-type, homologous recombination and random insertion genotypes, respectively. In the screening experiments for HR line, many RI lines are generated and primers, c and d, are used to distinguish HR from RI. Genotyping for Mpcle1 and Mptdr gene-targeting mutants (right). Three different primer sets (a-b, c-x, d-y) for each gene were used in genomic PCR to detect wild-type specific (yellow arrows) and mutant-specific (cyan arrows) products. Lanes (wt and mut) are for the PCR products from wild type (Tak-1) and mutant (Mpcle1-GT85 or Mptdr-GT400) DNA samples, respectively. (B) 14-day-old plants grown from gemmae for complementation test. (C) The antheridiophore morphology of MpCLE1 complementation line. See also Fig 4. (TIF) [file pgen.1007997.s004.tif]

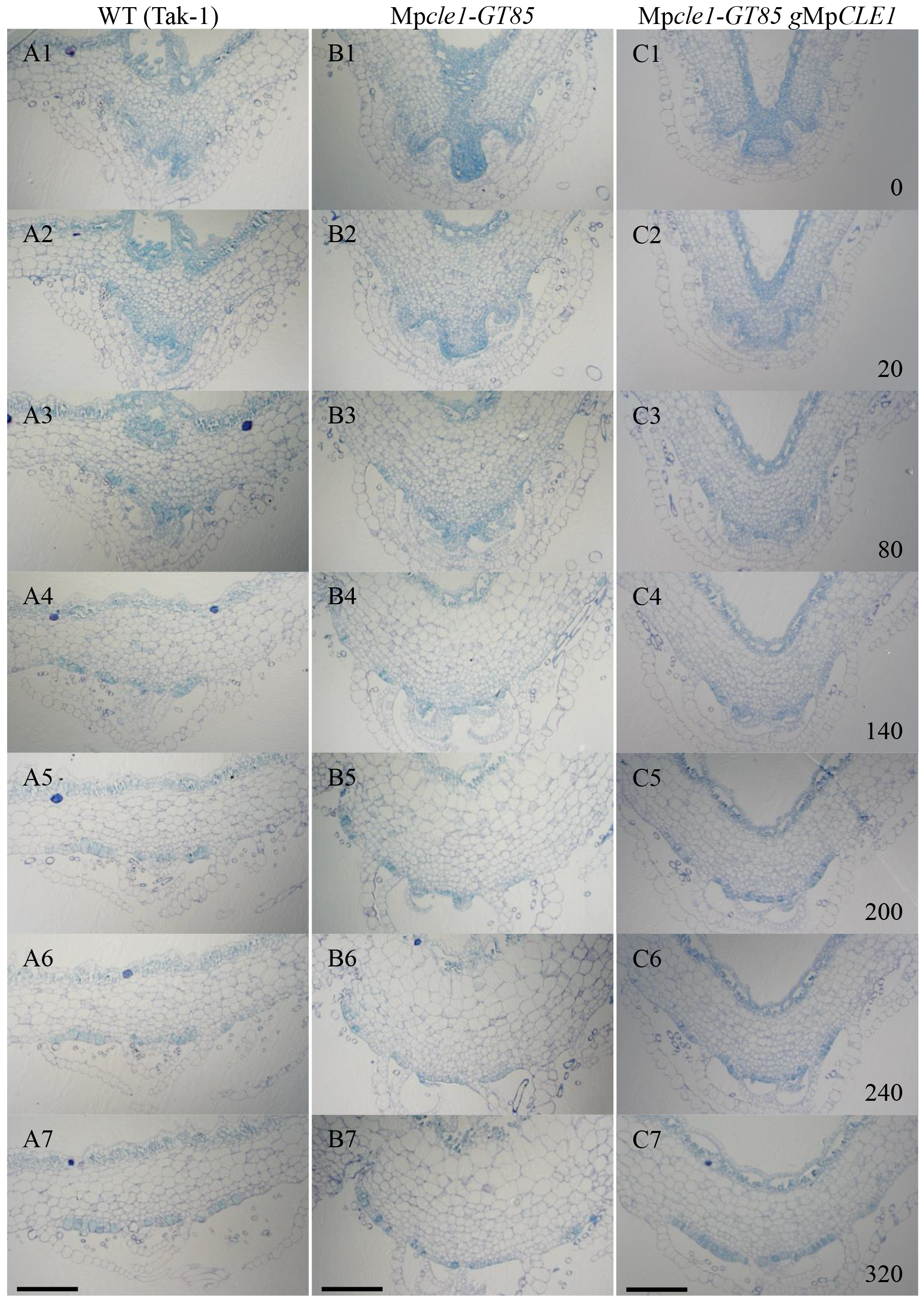

Supplement: S5 Fig — Consecutive transverse sections of apical notches in 14-day-old plants grown from gemmae. Comparison of WT (A1-A7), Mpcle1-GT85 (B1-B7) and a complementation line (C1-C7), with measurements as in Fig 3. The relative position (μm) is indicated at the right bottom corner of each panel. Scale bars = 200 μm. (TIF) [file pgen.1007997.s005.tif]

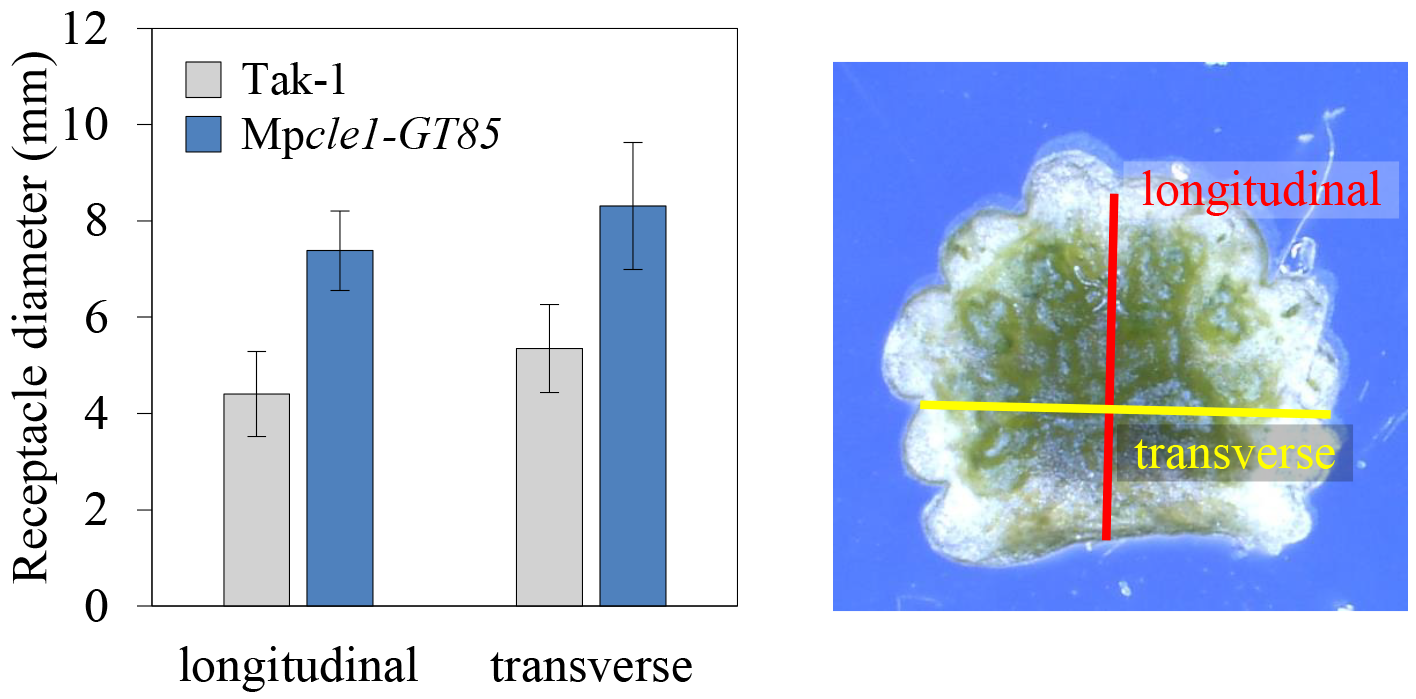

Supplement: S6 Fig — Diameters of receptacles in two axes (longitudinal and transverse as in right panel) are compared between Tak-1 (wild type) and Mpcle1-GT85 mutant. Data represents mean values ± s.d. (p<0.05 in Weltch’s t-test, n = 10–12). (TIF) [file pgen.1007997.s006.tif]

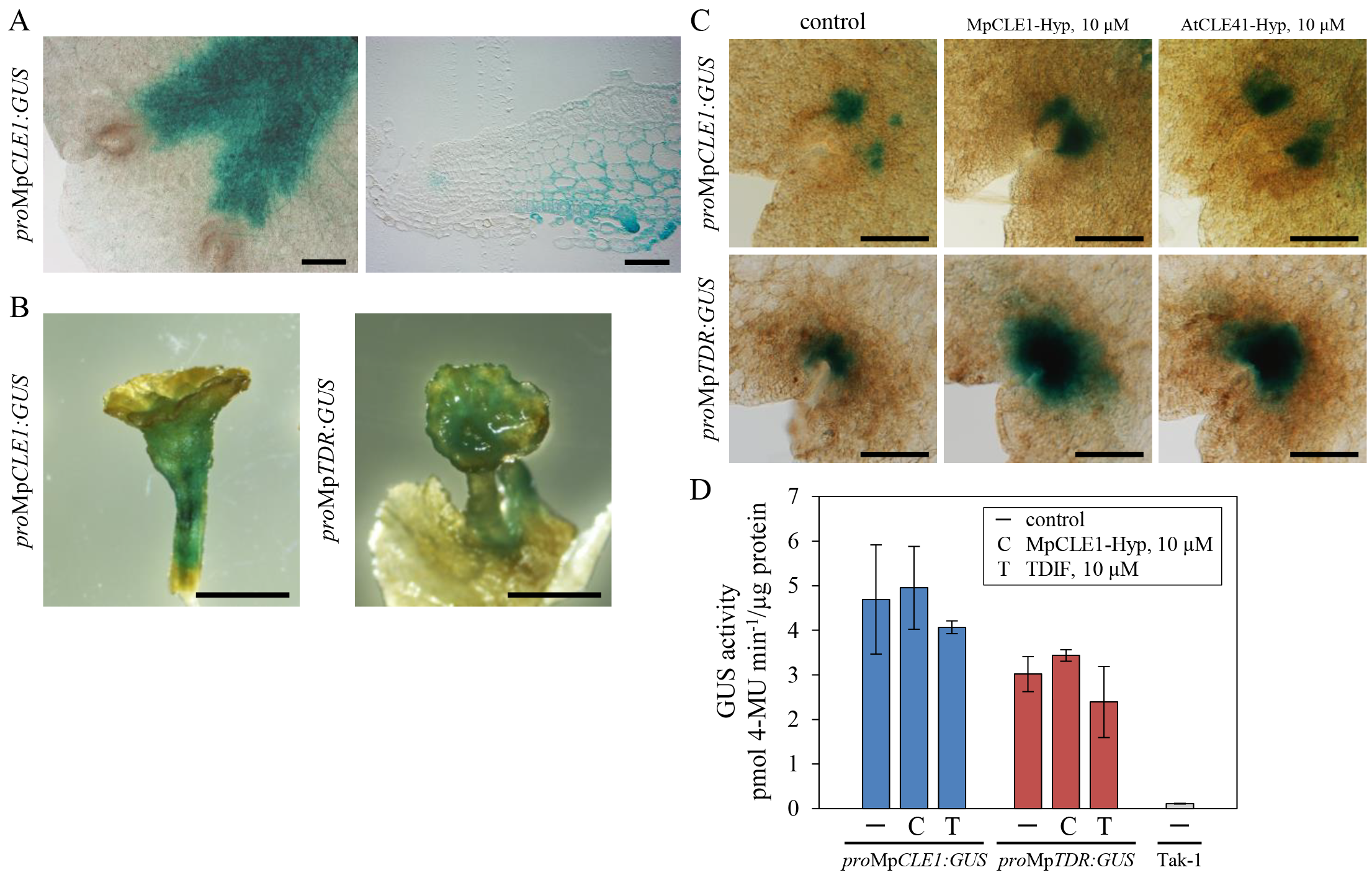

Supplement: S7 Fig — (A) Promoter-GUS assay for MpCLE1 in 10-day-old plants grown from gemmae. Whole mount sample (left) and longitudinal section (right) are shown. Note that GUS signals are detected within the meristem and along midrib. Scale bars indicate 500 μm (left) and 200 μm (right). (B) GUS activities in immature antheridiophores. Scale bars indicate 5 mm. (C) GUS activities in the apical notches of 5-day-old gemmalings grown with or without peptide as indicated above. Scale bars indicate 200 μm. (D) Fluorometric quantification of GUS activities on plants grown in the same conditions as (C). Data represent the mean values ± s.d. of 3 biological replicates. No significant differences were detected between peptide treatment and mock treatment for either proMpCLE1:GUS or proMpTDR:GUS (p>0.2 in Weltch’s t-test). (TIF) [file pgen.1007997.s007.tif]

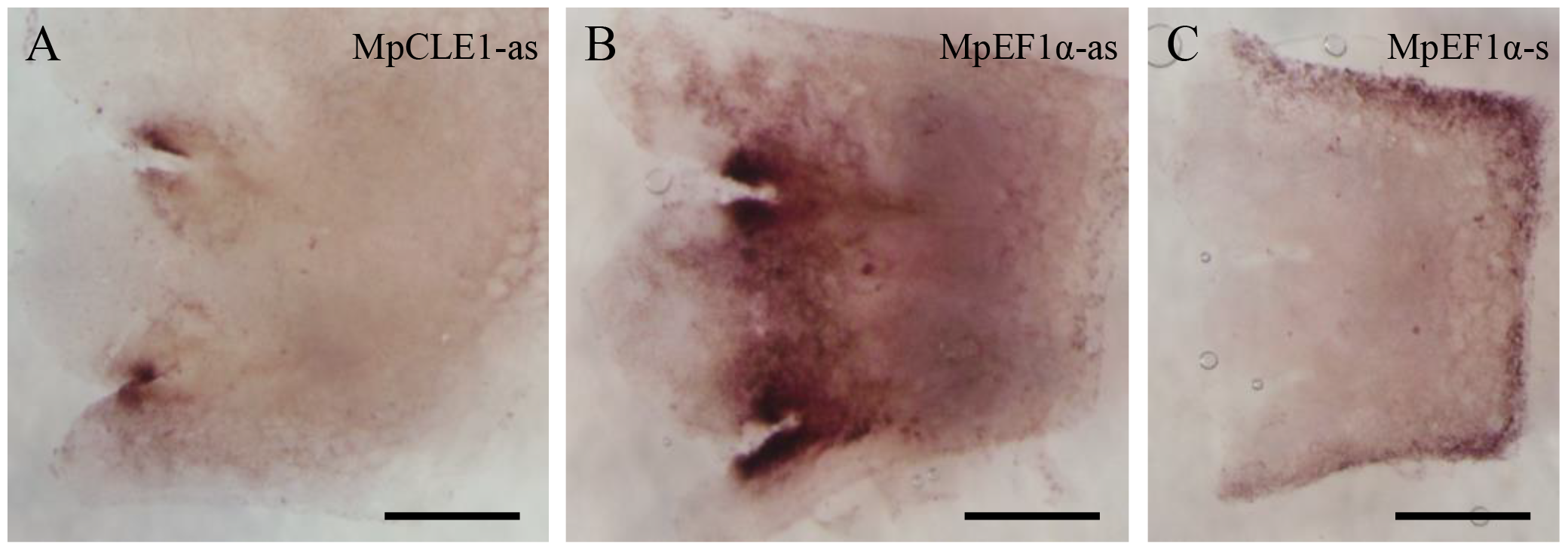

Supplement: S8 Fig — Whole mount in situ hybridization (WISH) assays in M. polymorpha thalli with probes as follows: MpCLE1-antisense in (A), MpEF1α-antisense in (B) and MpEF1α-sense in (C). Note that the MpEF1α probes were used for positive and negative controls. Scale bars = 500 μm. (TIF) [file pgen.1007997.s008.tif]
